# Potential mogamulizumab-associated inflammatory bowel disease in cutaneous T-cell lymphoma management

**DOI:** 10.1016/j.jdcr.2024.09.021

**Published:** 2024-10-12

**Authors:** Gabriela Blanchard, Maël Blanchard, Pauline Bernard, Jacqueline Doms, Begonia Cortés, Serge Boulinguez, Emmanuella Guenova

**Affiliations:** a Division of Dermatology and Venereology, University Hospitals of Geneva, Geneva, Switzerland; b Division of Dermatology and Venereology, Lausanne University Hospital (CHUV), Lausanne, Switzerland; c Faculty of Biology and Medicine, University of Lausanne, Lausanne, Switzerland; d Immunology and Allergy Division, Department of Medicine, Centre Hospitalier Universitaire Vaudois (CHUV), Lausanne, Switzerland; e Department of Dermatology, Centre Hospitalier Universitaire Toulouse, Toulouse, France; f Department of Dermatology, Hospital 12 de octubre, Medical School, University Complutense, Madrid, Spain; g University Institute and Clinic for Immunodermatology, Medical Faculty, Johannes Kepler University, Linz, Austria

**Keywords:** CCR4, CTCL, cutaneous T-cell lymphoma, IBD, inflammatory bowel disease, mogamulizumab, mycosis fungoides, Sézary syndrome

Mogamulizumab, a monoclonal antibody targeting C-C chemokines receptor 4 via antibody-dependent cellular cytotoxicity, has gained a rapidly growing importance in the management of cutaneous T-cell lymphomas (CTCL). Overall, mogamulizumab has a favorable side effect profile. However, some rare adverse events might not have been observed yet due to its relatively recent introduction.

We report on 2 CTCL patients who developed mogamulizumab-induced inflammatory bowel disease within 3 months of initiating mogamulizumab treatment. While the association with mogamulizumab is not directly proven, this observation underscores the need for ongoing monitoring and research into potential adverse effects.

## Case 1

**Fig 1:**
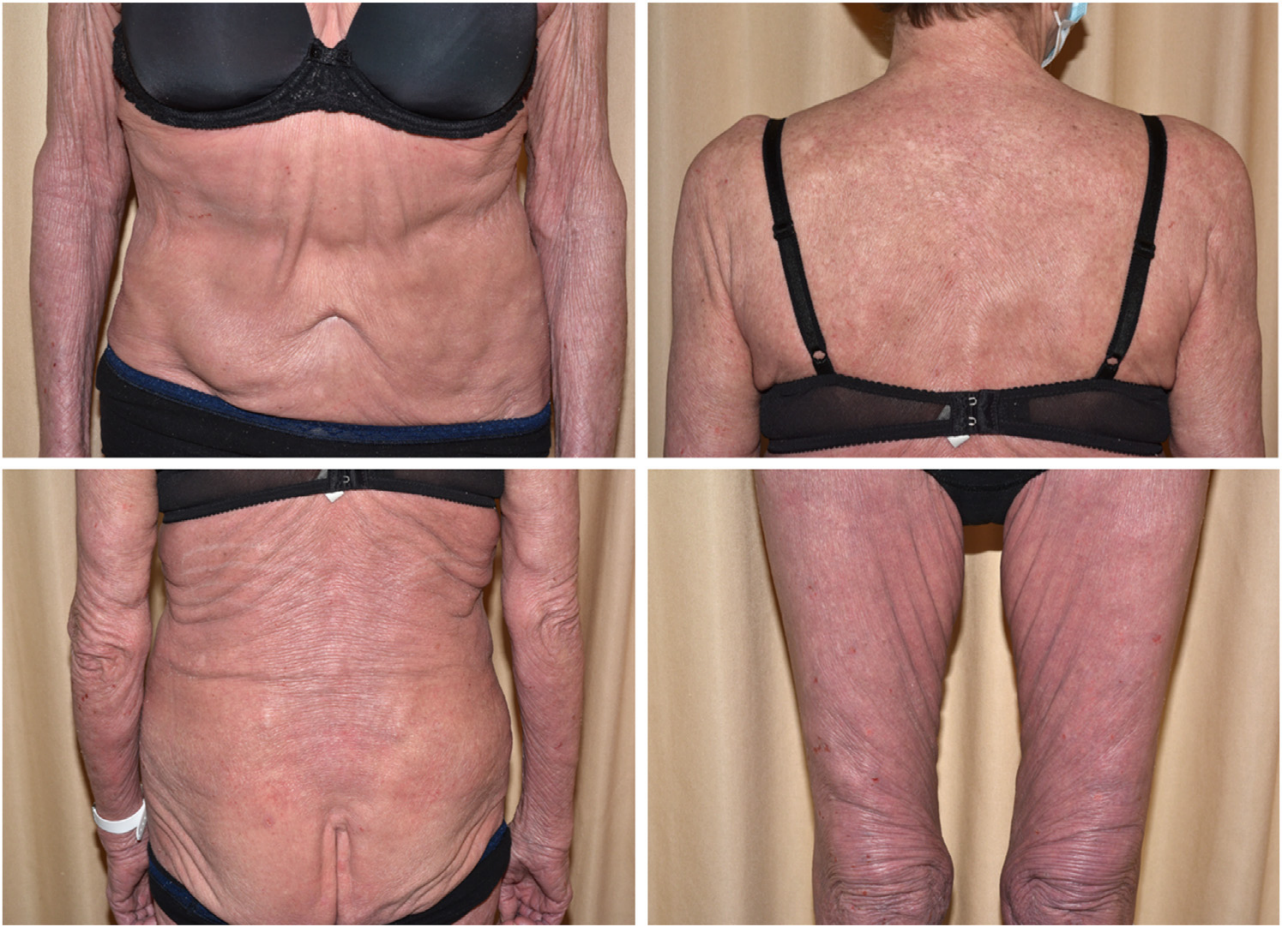
Clinical presentation with erythroderma following progression after 2 years of extracorporeal photopheresis and methotrexate in patient 1.

**Fig 2:**
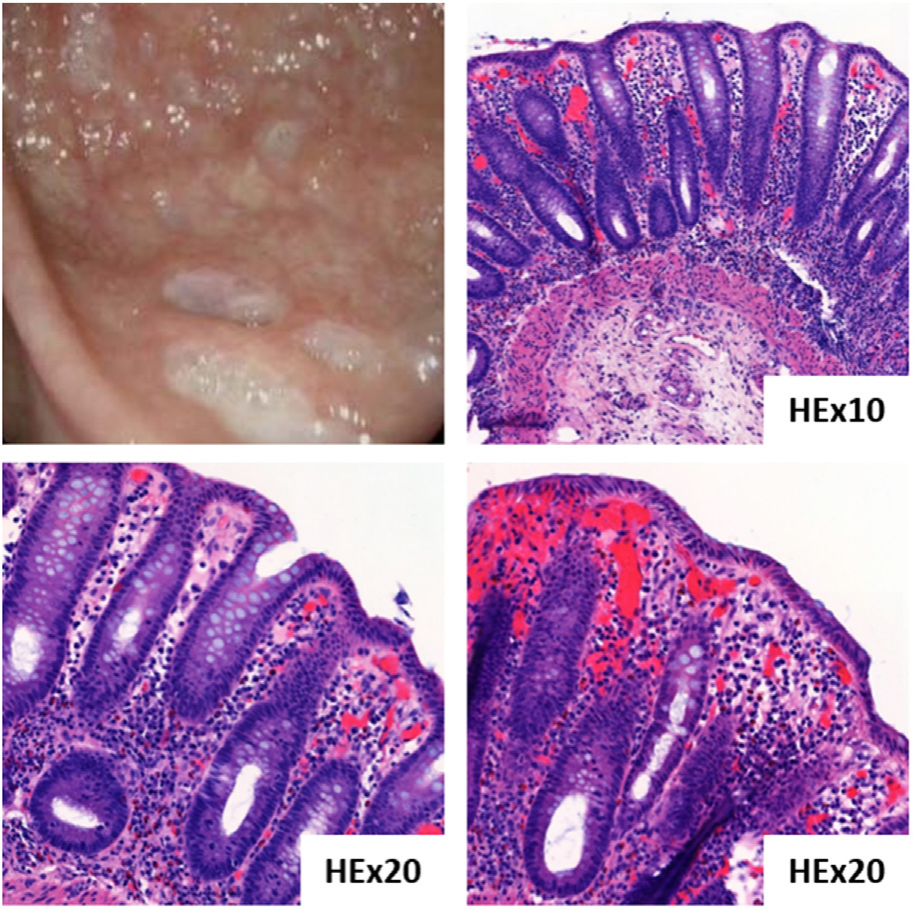
Endoscopy showing ulcerative pancolitis with histology compatible with a drug-induced colitis in patient 1.

An 87-year-old female was referred to our clinic for the management of stage IVA1 (T4NxM0B2) Sézary syndrome, which had progressed after 2 years of combined treatment with methotrexate and extracorporeal photopheresis (Fig 1). Mogamulizumab was initiated, resulting in complete skin and blood remission after only 3 cycles. After 3 months of mogamulizumab following the standard dosing (mogamulizumab 1 mg/kg intravenously on a weekly basis for the first 28-day cycle, then on days 1 and 15 of subsequent cycles), the patient developed severe, progressively worsening diarrhea. An infectious cause was ruled out. No additional treatments were introduced concomitantly with mogamulizumab. The patient had no known history of autoimmune disorder, smoking, or recent use of nonsteroidal anti-inflammatory drugs or proton pump inhibitors. The chronology of events raised the suspicion of an immune-related etiology associated with mogamulizumab treatment. Endoscopic evaluation revealed ulcerative pancolitis with histology indicating drug-induced colitis (Fig 2). Despite mogamulizumab’s discontinuation, the symptoms persisted, leading to a diagnosis of severe grade 4 mogamulizumab-induced colitis. Systemic corticotherapy was initiated, followed by selective immunosuppressive therapy with vedolizumab, yielding a limited effect. Despite its potential to worsen CTCL, treatment with monoclonal anti-tumor necrosis factor alpha (anti-tumor necrosis factor) antibody infliximab was started, resulting in a favorable intestinal response. Initially, the patient maintained complete clinical and blood response 1 year after stopping mogamulizumab. Following infliximab initiation, a worsening of skin and blood tumor burden was observed. Extracorporeal photopheresis was reinitiated, and the patient has since remained in complete remission.

## Case 2

A 64-year-old female with folliculotropic mycosis fungoides stage IIB (T3N0M0B1) previously treated with phototherapy, acitretin, methotrexate, pegylated interferon alfa, extracorporeal photopheresis, bexarotene, chlormethine gel, and total skin electron beam therapy was started on mogamulizumab with a good clinical response. After a few weeks of treatment (4 cycles of mogamulizumab), the patient developed rectal syndrome, rectorrhagia, and chronic diarrhea. The symptoms persisted despite mogamulizumab’s discontinuation. Endoscopy confirmed colitis with histology suggestive of inflammatory bowel disease. Mesalazine was stopped because of side effects. Systemic corticotherapy was initiated with a good intestinal response, followed by a recent introduction of vedolizumab as a corticosteroid-sparing agent. For the time being, the patient maintains a favorable skin and blood response.

Mogamulizumab safety evaluations have shown a very favorable profile compared to most chemotherapeutic agents used in CTCL management, with mainly mild infusion-related reactions, cutaneous drug rash, drug-related diarrhea, and fatigue. Diarrhea concerned more than 20% of patients but was generally mild and well-tolerated, not requiring any discontinuations of mogamulizumab in the Mogamulizumab versus vorinostat in previously treated cutaneous T-cell lymphoma (MAVORIC) trial.^1^^,^^2^ In such cases, the improvement of diarrhea is expected upon mogamulizumab withdrawal. Nevertheless, both of our patients experienced persistent symptoms despite the cessation of mogamulizumab with histological findings consistent with inflammatory bowel disease. In Europe, the annual incidence of ulcerative colitis varies from 0.6-24.3 per 100,000 and from 0.3-12.7 per 100,000 for Crohn’s disease.^3^ It cannot be excluded that mogamulizumab may have contributed to the development and/or unmasking of an autonomous autoimmune inflammatory colitis.

Due to its depleting effect on both tumor cells but also regulatory T cells expressing C-C chemokines receptor 4, mogamulizumab was identified as a potential autoimmune disease inducer or reactivator as previously shown in adult T-cell leukemia/lymphoma patients.^4^ Consequently, patients with known autoimmune disease were excluded from the MAVORIC trial. Autoimmune complications were reported in 6 out of 319 patients receiving mogamulizumab, including myocarditis, hepatitis, myositis and polymyositis, pneumonitis, and a rare variant of Guillain-Barré syndrome known as Miller-Fisher syndrome.^1^^,^^2^^,^^5^ Additionally, cases of vitiligo and alopecia areata associated with mogamulizumab treatment have been reported, and both have been suggested as positive predictors of treatment response.^6^^,^^7^ In a French cohort of 21 patients treated with mogamulizumab, 2 developed confirmed autoimmune disease. The first patient experienced autoimmune hepatitis, alopecia areata, vitiligo, and mild autoimmune thyroiditis, while the second patient developed autoimmune hemolytic anemia. Both patients experienced an ongoing complete skin and blood response for at least 3 and 2 years, respectively. This sustained complete remission has been attributed to the activation of cytotoxic antitumor T cells via the depletion of C-C chemokines receptor 4-positive regulatory T cells.^8^ Due to this mechanism, pretransplantation mogamulizumab treatment is also considered a risk factor of severe graft-versus-host disease after allogeneic stem cell transplant, and mogamulizumab should not be administered within 3 months preceding transplant.^2^^,^^5^^,^^9^ This mechanism may also explain the prolonged remission observed in our first patient, further supporting the proposition that mogamulizumab-associated autoimmunity might indicate a favorable CTCL prognosis.

The treatment of enterocolitis was based on the analogy to the management of toxicities from immunotherapy according to the European Society for Medical Oncology.^10^ Consistently, both patients received systemic corticotherapy, followed by anti-α_4_β_7_ integrin monoclonal antibody vedolizumab. In the first patient, the clinical response was insufficient, requiring the use of the tumor necrosis factor-alpha blocker infliximab. In accordance with previous reports,^11^ treatment with tumor necrosis factor-alpha blocker led to a gradual skin and blood worsening. This relapse was however effectively managed solely with the reintroduction of extracorporeal photopheresis, which had previously proved insufficient in our patient.

In conclusion, the emergence of mogamulizumab as a novel therapeutic option has revolutionized CTCL management. Its recent implementation into clinical practice still requires careful monitoring. Despite the generally favorable safety profile, mogamulizumab has the potential to cause severe autoimmune complications, even in patients without a clear prior history of autoimmune conditions. Nevertheless, these manifestations might be associated with a good CTCL response. These cases illustrate a rare but significant adverse effect of mogamulizumab treatment, manifesting as drug-induced colitis. Clinicians should be aware of this potential risk and monitor patients closely for any signs of gastrointestinal distress. Early recognition and appropriate management, including discontinuation of mogamulizumab and initiation of appropriate immunosuppressive therapy, are crucial to ensure patient safety. Further research is needed to fully comprehend the long-term safety profile of mogamulizumab to mitigate its adverse effects as well as to better understand the association between autoimmunity and long-term remission following mogamulizumab treatment.

## Conflicts of interest

Dr Guenova received honoraria and/or grant support from Mallinckrodt, Helsinn, Takeda Pharmaceuticals, Recordati Rare Diseases, Novartis, Sanofi, Stemline Therapeutics, and Kyowa Kirin. Dr Maël Blanchard received honoraria from Kyowa Kirin. Dr Boulinguez received honoraria and/or is a scientific committee member for Recordati, Pierre Fabre Dermatologie, Sanofi, Amgen, UCB, and Novartis. Drs Cortés, Doms, Bernard, and Gabriela Blanchard have no conflicts of interest to declare.
